# Limitation of Non-Beneficial Interventions and their Impact on the Intensive Care Unit Costs

**DOI:** 10.2478/jccm-2023-0028

**Published:** 2023-11-14

**Authors:** Sotiria Koutsouki, Dimitrios Kosmidis, Eva-Otilia Nagy, Alexandra Tsaroucha, Georgios Anastasopoulos, Ioannis Pnevmatikos, Vasileios Papaioannou

**Affiliations:** General Hospital of Kavala, Kavala, Greece; Nursing Department, International Hellenic University, Didymoteicho, Greece; General Hospital of Kavala, Kavala, Greece; Postgraduate program on Bioethics, Laboratory of Bioethics, Medical School, Democritus University of Thrace, Alexandroupolis, Greece; Medical Informatics Laboratory, Democritus University of Thrace, Alexandroupolis, Greece; Faculty of Medicine, University of Cyprus, Lefkosia, Cyprus; Faculty of Medicine, Democritus University of Thrace, Alexandroupolis, Greece

**Keywords:** non-beneficial interventions, end-of-life, cost of care, intensive care units

## Abstract

**Introduction:**

Using a plan to limit non-beneficial life support interventions has significantly reduced harm and loss of dignity for patients at the end of life. The association of these limitations with patients’ clinical characteristics and health care costs in the intensive care unit (ICU) needs further scientific evidence.

**Aim of the study:**

To explore decisions to limit non-beneficial life support interventions, their correlation with patients’ clinical data, and their effect on the cost of care in the ICU.

**Material and Methods:**

We included all patients admitted to the general ICU of a hospital in Greece in a two-year (2019–2021) prospective study. Data collection included patient demographic and clinical variables, data related to decisions to limit (withholding, withdrawing) non-beneficial interventions (NBIs), and economic data. Comparisons were made between patients with and without limitation decisions.

**Results:**

NBIs were limited in 164 of 454 patients (36.12%). Patients with limitation decisions were associated with older age (70y vs. 62y; p<0,001), greater disease severity score (APACHE IV, 71 vs. 50; p<0,001), longer length of stay (7d vs. 4.5d; p<0,001), and worse prognosis of death (APACHE IV PDR, 48.9 vs. 17.35; p<0,001). All cost categories and total cost per patient were also higher than the patient without limitation of NBIs (9247,79€ vs. 8029,46€, p<0,004). The mean daily cost has not differed between the groups (831,24€ vs. 832,59€; p<0,716). However, in the group of patients with limitations, all cost categories, including the average daily cost (767.31€ vs. 649.12€) after the limitation of NBIs, were reduced to a statistically significant degree (p<0.001).

**Conclusions:**

Limiting NBIs in the ICU reduces healthcare costs and may lead to better management of ICU resource use.

## Introduction

Intensive care units (ICUs) have contributed to increasing the survival of critically ill patients and prolonging the dying process. Providing futile treatment consumes scarce healthcare resources, delays the care of other patients, and can prolong suffering and deprive patients of dignity [1, 2, 3]. Avoiding ineffective treatment has led the international scientific community to establish practices of non-beneficial life support interventions, marking the transition from curative to palliative care [4, 5]. Using a plan to limit non-beneficial interventions (NBIs) reduces patient harm at the end of life, and its early adoption has been associated with fewer NBIs, less perceived suffering, and loss of dignity [6,7].

In addition, with the steady increase in life expectancy in many countries, the financial impact on health systems will be enormous, and the use of ICU resources will be valuable [8]. In the European Union, it is projected that 24.4 million people will be over 85 years of age in 2040. Rates of admission of elderly patients to the ICU accounted for 15% of ICU admissions, raising challenges in managing admitted patients and end-of-life decision-making [9].

Although capturing the economic impact of changing standards of care in ICUs is complex, the need for more studies to measure the actual cost of care is urgent [10]. Healthcare professionals and policymakers will need results from economic analyses to make better-informed decisions about allocating resources to healthcare [11]. In this context, studies on cost savings in ICUs based on alternative models of care have already started to be documented in the international literature. Bouttell et al. concluded that implementing a plan to limit NBIs for all expected deaths in Scottish hospital ICUs would save $3.1 million [12]. In the study by Chin-Yee et al., palliative care over full support in the ICU was an independent factor in reducing costs in older patients [13]. However, overusing aggressive care and underusing palliative care at the end of life are common in high and low-income countries [14]. Although there has been an international emphasis on research into issues related to the limitation of NBIs, studies investigating their impact on the cost of care for these patients have been rare. This study investigated decisions to limit non-beneficial life support interventions, their correlation with patients’ clinical data, and their impact on ICU care costs.

## Material and Methods

The study was a prospective single center observational study conducted in the general adult ICU of the General Hospital of Kavala, Greece. The study was approved by the hospital’s Ethics committee (no. 2o/14/11-6-2019). All patients 18 years or older admitted to the participating ICU were eligible. The data collection period was two consecutive years (from 1/7/2019 to 30/6/2021).

The demographic and clinical characteristics of the patients, as well as ICU data, were collected by daily recording in a specialized database maintained in the ICU. Cost data were obtained from the hospital’s information system and data from the above database.

Data collection included patient demographic and clinical data (age, sex, reason for admission, disease severity, length of stay), outcome (ICU mortality and 60-day mortality), variables related to clinical decisions to limit NBIs (day and type of decision, length of ICU stay before and after the decision, type of interventions that limited) and variables related to economic data (variable and fixed costs).

All decisions to limit non-beneficial interventions were at the discretion of the treating physician(s) on each shift based on international recommendations. They were validated by the entire medical team at their daily meeting. A nurse extracted information about limitations from clinical sessions and medical records and entered them into the ICU database. A non-beneficial intervention was defined as an intervention that did not achieve stabilization or reversal of the patient’s clinical condition. Non-beneficial interventions included administration of vasoactive/inotropic drugs, mechanical ventilation, noninvasive ventilation, and renal function replacement therapies. The decision to limit NBIs included the application of withholding (WH) or withdrawal (WD) of one or more interventions. WH was the decision not to initiate or increase one or more interventions, and WD was to discontinue one or more.

Disease severity was calculated using the APACHE IV scale in the first 24 hours after admission. The standardized Mortality Ratio (SMR) was based on APACHE IV predicted death rate.

The microcosting (bottom-up) methodology with a combination of attributable (top-down) was used to calculate costs [15, 16]. Variable costs included costs that depended on the number of inpatients and related to direct patient care (imaging and laboratory tests, drugs, patient consumables). Imaging and laboratory tests were calculated based on the current government prices. Fixed direct costs were defined as those independent of the number of inpatients and related to direct patient care (staff salaries). Fixed indirect costs were those independent of the number of inpatients and do not relate to indirect patient care (cleaning materials, linen, technical materials, stationery, purchase and maintenance of equipment, hospital support services, and depreciation of fixed capital). Direct variable costs were calculated using the microcosting (bottom-up) method by counting each activity performed (imaging and laboratory tests, drugs, consumables) at the patient level. However, the costs of the variable sub-categories (drugs, consumables, and tests) of the daily costs were calculated cumulatively for each patient and then allocated according to the length of stay.

Furthermore, for patients with NBIs, limitation decisions were calculated before and after the decision date and then distributed according to the days of stay of each patient, before and after the decision date. In contrast, indirect fixed costs (hospital support services, purchase and maintenance of equipment) were calculated using the top-down costing method for the specified period and allocated proportionally to each patient’s length of stay (number of days). Direct fixed costs were calculated by counting the monthly salary for each personnel of the ICU, summarized, and then distributed according to the length of stay of each patient. The total cost per patient was obtained by summing each patient’s three categories (variable, fixed direct, and fixed indirect costs). All costs were calculated based on the prices of the starting year of the study in the current European common currency (euro).

### Statistical analysis

Categorical variables were expressed in absolute frequencies and percentages, while continuous variables were expressed in means, standard deviations, median, and interquartile ranges. The normality assumption was evaluated using the Kolmogorov-Smirnov criterion. Parametric and nonparametric criteria (Chi-squared test, Mann-Whitney U test) were used to correlate the groups of patients with and without limitation decisions and the differences in demographic and clinical characteristics. Wilcoxon signed-rank test was used to conduct a paired difference test of repeated measurements (costs before and after limitation decisions). In all cases, 5% or less (p-value<0.05) was statistically significant. Statistical analysis was performed using the Statistical Package for the Social Sciences (IBM SPSS) version 25.0.

## Results

**Fig.1.**
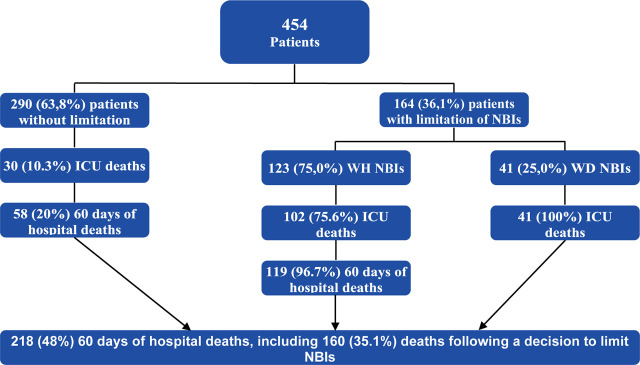
Patient flow chart

Decisions to limit NBIs were made in 164 (36.12%) patients (Figure 1). The baseline characteristics of the patients were described in detail in Tables 1, 2.

**Table 1.** Patients’ characteristics

**Patients’ characteristics**	**All patients(N=454)**	**Patients with no limitation of NBIs (N=290)**	**Patients with limited NBIs (N=164)**	**P-value**
Gender–Male, n(%)	304 (67,0)	195 (67,2)	109 (66,5)	0,866
Age, median (IQR)	65 (21)	62 (22)	70 (14)	0,000
APACHE IV, median (IQR)	58 (35)	50 (31)	71 (42)	0,000
APACHE IV PDR, median (IQR)	28,4 (36,6)	17,35 (27,2)	48,9 (31,9)	0,000
LOS, median (IQR)	5 (12)	4,5 (11)	7 (13)	0,004

**Reason for admission n (%)**				
Surgery	99 (21,81)	92 (31,7)	7 (4,3)	0,001
Acute respiratory failure (COVID-19)	95 (20,93)	41 (14,1)	54 (32,9)	0,001
Acute Respiratory Failure	59 (13)	41 (14,1)	18 (11,0)	0,385
Traumatic brain injury	43 (9,47)	22 (7,6)	21 (12,8)	0,094
Nontraumatic CNS injuries	43 (9,47)	25 (8,6)	18 (11,0)	0,409
Cardiac arrest	33 (7,27)	11 (3,8)	22 (13,4)	0,001
Sepsis	25 (5,51)	11 (3,8)	14 (8,5)	0,052
Multiple trauma	18 (3,96)	17 (5,9)	1 (0,6)	0,005
Multiple trauma (with TBI)	14 (3,08)	9 (3,1)	5 (3,0)	0,607
Other	25 (5,51)	21 (7,2)	4 (2,4)	0,033

**Comorbidities at ICU admission n (%)**				
Chronic pulmonary disease	72 (15,9)	36 (12,4)	36 (22,0)	0,011
Cardiovascular disease	134 (29,5)	80 (29,5)	54 (32,9)	0,412
Chronic kidney disease	26 (5,7)	8 (2,8)	18 (11,0)	0,001
Hypertension	170 (37,4)	96 (33,1)	74 (45,1)	0,012
Disorders of thyroid gland	31 (6,8)	20 (6,9)	11 (6,7)	0,553
Diabetes mellitus	88 (19,4)	46 (15,9)	42 (25,6)	0,014
Cancer	39 (8,6)	26 (9,0)	13 (7,9)	0,862
Obesity	22 (4,8)	11 (3,8)	11 (6,7)	0,177
Other	186 (41,0)	128 (44,1)	58 (35,4)	0.074

ICU mortality, n (%)	173 (38,1)	30 (10,3)	143 (87,2)	0,000
60 days hospital mortality, n (%)	218 (48,0)	58 (20,0)	160 (97,6)	0,000
SMR, 95% Confidence Interval (CI)	1,18 (0.88–1.58)	0,47 (0.28–0.78)	1,74 (1.55–2.05)	
Variable costs per patient, mean (SD)	3111,87 (4306,06)	3047,71 (4661,21)	3226,02 (3599,26)	0,017
Fixed direct costs per patient, mean (SD)	4263,75 (5029,56)	3969,38 (5010,92)	4798,06 (5047,95)	0.004
Fixed indirect costs per patient, mean (SD)	1087,44 (1282,75)	1012,36 (1278,00)	1223,72 (1287,44)	0,004
Total cost per patient, mean (SD)	8467,85 (10179,37)	8029,46 (10490,42)	9247,79 (9582,97)	0,004
Average daily cost per patient, mean (SD)	832,10 (163,82)	832,59 (159,46)	831,24 (171,80)	0,716

NBIs: Non Beneficial Interventions; APACHE: Simplified Acute Physiology Score; PDR: predicted death rate; LOS: length of stay; SMR: Standardised Mortality Ratio; IQR: Interquartile Range; SD: Standard Deviation; CNS: central nervous system; TBI: Traumatic brain injury.

**Table 2.** Length of stay in ICU of patients with limitation of NBIs

**LOS (in ICU)**	**Withholding NBIs**		**Withdrawing NBIs**	
	**Before decision**	**After decision**	**Before decision**	**After decision**
N	123		41	
Mean	8,93	3,94	4,12	2,49
SD	10,94	4,44	3,68	2,94
Median	6	2	3	2
IQR	12	4	4	3
Min	1	1	1	1
Max	64	27	15	17
Sum	1098	485	169	102

LOS: Length of stay; NBIs: Non-Beneficial Interventions

**Fig.2.**
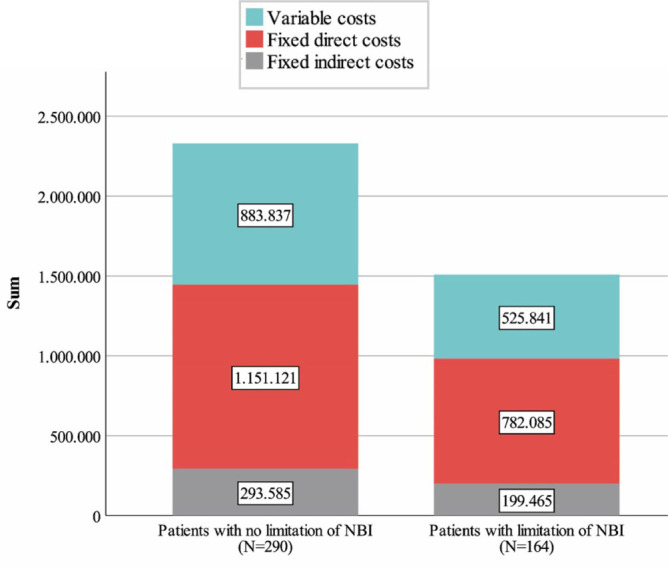
Total patients cost per category.

The NBIs most often limited were vasoactive drugs (78,0%) and invasive mechanical ventilation (57,9%) (Table 3). The total cost of all patients for the study period was 3.835.933,83€. Variable, direct, and indirect fixed costs accounted for 36.7%, 50.4%, and 12.8% of the total ICU costs, respectively. The total cost (Figure 2) of patients without limitation of NBIs was 2,328,543€, while for the patients with limitation of NBIs, it was 1,507,391€, a difference of 821,000€. The cost per patient was, both as a whole and in its categories (variable, fixed), higher in the group of patients with a decision to limit NBIs (Table 1). All cost categories, including the average daily cost after the limitation of NBIs, were reduced to a statistically significant degree (p<0.001) (Table 4). The total cost of the patients with the limitation of NBI was 1,105,262.13€ before the decision, while after the decision, it was 402,129.22€.

**Table 3.** Type of Limited NBIs

**Limited NBIs (n, %)**	**(N=164)**
Vasoactive drugs	128 (78,0)
Invasive mechanical ventilation	95 (57,9)
Noninvasive mechanical ventilation	27 (16,5)
Continuous renal replacement techniques	17 (10,4)

NBIs: Non-Beneficial Interventions

**Table 4.** Cost category per patient before and after the limitation of NBIs

**Cost category per patient**	**Mean(SD)**	**P value**
Variable cost (before)	2666,13 (3306,89)	0,001
Variable cost (after)	556,6 (735,3)	

Fixed direct costs (before)	3269,52 (4173,84)	0,001
Fixed direct costs (after)	1514,76 (1759,64)	

Fixed indirect costs (before)	833,86 (1064,5)	0,001
Fixed indirect costs (after)	366,33 (448,78)	

Total cost (before)	6769,53 (8331,59)	0,001
Total cost (after)	2457,7 (2652,68)	

Daily cost (before)	767,31 (405,52)	0,001
Daily cost (after)	649,12 (243,14)	

NBIs: Non-Beneficial Interventions; SD: Standard Deviation

## Discussion

Doctors and nurses in the ICU are constantly faced with the prospect of patient death and often the limitation of NBIs. The most common limitation of treatments in the study sample was withholding of NBIs, followed by withdrawal, which is consistent with the results of previous studies, particularly those from southern Europe, and probably consistent with the cultural background of these countries [17,18]. The positive association between increased age and limited NBIs argues that age was part of the clinical assessment and consideration of other factors that led to these decisions [19, 20, 21].

Patients with limitations of NBIs had a higher disease severity score and a worse prognostic probability of death than patients without limitations of NBIs, and this has been documented by other researchers [5, 20, 21, 22, 23]. Although scoring systems such as APACHE are helpful, they may increase the likelihood of self-fulfilling prophecy by health professionals [24]. The relatively extended length of stay in the ICU before the decision is made, coupled with the increased rate of withholding of NBIs, partially invalidates the self-fulfilling prophecy theory.

In the present study, the length of stay in the ICU of patients with NBIs was longer than that of patients without NBIs [17, 22, 23].

The interpretation of this longer duration is probably due to the exhaustion of treatment options and may partly explain the delay in decisions, especially of WH of NBIs. In contrast, the length of stay and time to decision in patients with withdrawal were shorter. Physicians may have taken imminent death into account, resulting in early avoidance of aggressive interventions that were likely to prolong death and cause suffering. Physicians may perceive the length of stay in the ICU as a prognostic factor when considering making changes to therapeutic targets and indications for treatment [25]. It likely indicates a severe underlying disease, the failure of treatment or lack of treatment options in the context of the increased severity of the disease, or even the fact that these patients were admitted to the ICU for reasons beyond medical necessity. The sunk cost fallacy could also play an essential role in the admission decisions of patients with a low probability of survival. According to this logic, as long as ICU beds are available, they can be used regardless of the patient’s survival prospects. This phenomenon has been referred to in the literature as the difficulty of parting with previously invested financial resources, even if these investments’ outcomes do not meet expectations [26, 27].

Our study found that limitations of NBIs were applied most frequently in patients with medical rather than surgical conditions. The most significant proportion of patients with limitations of NBIs involved acute respiratory failure (COVID-19), cardiac arrest, and traumatic brain injury. The COVID-19 pandemic abruptly shifted the causes of admission for ICU patients by becoming the leading cause of admission for an extended period. The non-response of COVID-19 patients to treatment, combined with factors such as severity and age, may have contributed to the limitations of NBIs decisions. Similar results were found in the Flaatten study, where 37% of patients with COVID-19 had life support intervention limitations applied to them. Withholding was the most common limitation, and the 30-day mortality of patients with limitation of NBIs was 79% [28]. Other studies have reported medical causes of admissions as independent predictors of limitation or increased incidence rates [20, 21, 23]. In contrast to surgical patients in our study, limitation decision was low, probably mainly due to their conventional admission to the ICU for safety and controlled awakening [5, 11, 19, 22].

In the present study, as in previous ones, it is found that the limitations of NBIs are associated with a high rate of deaths in the ICU [17, 19, 29, 30]. The fact that 14% of patients survived ICU indicates that limitations of NBIs do not only occur at the end of life. Similarly, it has been reported in other studies that a proportion of patients survive in the ICU after NBIs limitation [17, 23, 31, 32]. These results demonstrate that the quality of remaining life will likely improve without aggressive interventions, as person-centered care persists and is best delivered in an ICU setting where patient/nurse ratios differ from those in a conventional hospital ward. Besides, caring for the “Other” (a term used for the suffering human being) is an ethical imperative for ICU doctors and nurses [33]. The high outcome death rate in patients with limitations indicates the need for early implementation of palliative care measures, especially for patients with extended stays or transfer to other structures capable of continuing it.

The SMR was also higher in patients with limitations of NBIs, which other researchers have noted [22]. The higher SMR is likely due to factors such as the admission of patients to the ICU with increased severity, the negative progression of underlying severe disease, and the development of complications. Although expected, the positive association of mortality and SMR with life support limitations suggests problems with their use as measures of ICU quality, and caution is needed in interpreting their results [34]. In addition, the timing of the COVID-19 pandemic influenced the results to some extent. Mortality prediction tools in COVID-19 patients may have inaccuracies, as disease severity scale scores developed before the pandemic may underestimate severity in these specific patient groups and need to be revised [35, 36].

The cost per patient, both as a whole and in its categories (variable and fixed), was higher in patients with limitations of NBIs than in the group without limitations. This result indicates an increased use of resources by patients with limitations. These results are consistent with other studies regarding the ICU patients’ group at the end of life [37, 38, 39, 40]. Costs were likely influenced by factors such as the reason for admission, disease severity, and inability to transfer to another setting of appropriate care. Reducing the use of ICU resources may be feasible in countries where intermediate units and hospital wards provide a high level of care. As a previous study mentioned, dedicated hospice inpatient units are potentially significant sources of bed days and cost savings [41]. The lack of specialized palliative care services in public hospitals in Greece and the lack of suitable alternative models of outpatient healthcare structures to transfer these patients place a heavy burden of end-of-life care on the ICU. Greece is among the countries facing a shortage of palliative care services, often home-based and insufficient to meet the population’s needs [42]. The average daily cost per patient found was 832€±163.82€, and these amounts are consistent with previous analyses of ICU costs in European countries [43, 44]. Interestingly, this study’s average daily cost per patient showed no differences between the groups. The fact that average daily cost per patient has no differences may be because, as other researchers have found, fixed costs burden the variation in average daily costs - largely dependent on the length of stay - and less by variable costs [ 45,46,47].

In the group of patients with limitations, the length of stay in the ICU after the decision decreased, as other researchers had mentioned [17].

The timing of the decision impacted cost variation, and the type of limitation (WH, WD) may also affect our result. Our results also support the findings in the literature that demonstrate the importance of time limitation. The study by Zhao et al. showed that half of the patients and their relatives made limitation of NBIs decision less than four days after admission to the ICU, resulting in an immediate reduction in length of stay and costs, revealing an overall reduction in ICU resource utilization and out-of-pocket costs for patients and their relatives [48]. Our study showed a reduction of 118€ in ICU daily costs. Buttrick et al. also found an average decrease in ICU charges of 68.929$ per patient with limitations of NBIs [49]. Bouttell et al. retrospectively cost the limitation of NBIs and found a reduction in hospital costs [12]. However, cost comparisons are difficult because each study uses different calculation methods. Nevertheless, involving palliative care in critically ill patients has been shown to reduce resource overuse and length of stay in hospitals [50, 51, 52, 53].

Shifting care goals from aggressive treatment to comfort care reduced variable and fixed costs in the ICU. According to the results, the total cost of all patients after the limitation was 402,129€. If these patients had been transferred to other care structures after the decision had been taken, these costs would have been avoided. For example, this opportunity cost could have been used to pay the nursing staff in this ICU for one year. At the same time, 587 days of ICU care could have been freed up for other patients with a better survival prospect, as other researchers have also pointed out [41, 54].

These data reveal significant potential cost savings and overall better management of ICU resource utilization. Our study provides essential data on the limitations of NBIs’ impact on ICU costs and the potential to avoid such costs. Additionally, our study highlights a lack of care structures for patients nearing the end of life. It underscores the need to create such structures or redefine the role of the ICU to incorporate timely specialized palliative care services. Future healthcare planning decisions should be based on these estimates.

### Limitations

The present study has several limitations. The study was conducted in a single ICU, which limits the findings’ generalizability, as the specific unit’s care patterns may influence these. The study period coincided with the pandemic outbreak, which may have influenced the results as a proportion of the sample involved patients with COVID-19. The hospitalization of these patients was new to the physicians and nurses in the ICU, and it may have affected the making and timing of containment decisions, costs, and the care of these patients. Also, the reasons that led to the decisions had not been recorded since it was not the main object of our study. We did not record criteria or details about care delivery after limitations. Although no specific palliative care protocol was implemented, care was based on the general principles of palliative care and the principles of humanitarian care that apply in the ICU.

## Conclusion

Our study showed that limiting NBIs in the ICU reduces healthcare costs. The emphasis on identifying cost-effective ways to deliver quality end-of-life care must remain at the forefront of economic and social consciousness. Decisions to limit NBIs in ICU and their impact on ICU resources merit further research as they affect ICU health professionals, the health economy, and society. Education, redesigning clinical protocols, reallocating resources, and establishing national policies are essential to improve the quality of care and maintain dignity at the end of life.
